# IP-Dip-Based SPR Structure for Refractive Index Sensing of Liquid Analytes

**DOI:** 10.3390/nano11051163

**Published:** 2021-04-29

**Authors:** Petra Urbancova, Dusan Pudis, Matej Goraus, Jaroslav Kovac

**Affiliations:** 1 Department of Physics, Faculty of Electrical Engineering and Information Technology, University of Zilina, Univerzitna 1, 01026 Zilina, Slovakia; pudis@fyzika.uniza.sk (D.P.); goraus@fyzika.uniza.sk (M.G.); 2 Institute of Electronics and Photonics, Faculty of Electrical Engineering and Information Technology, Slovak University of Technology in Bratislava, Ilkovicova 3, 81219 Bratislava, Slovakia; jaroslav_kovac@stuba.sk

**Keywords:** surface plasmon resonance, 2D grating structure, refractive index sensing

## Abstract

In this paper, we present a two-dimensional surface plasmon resonance structure for refractive index sensing of liquid analytes. The polymer structure was designed with a period of 500 nm and prepared in a novel IP-Dip polymer by direct laser writing lithography based on a mechanism of two-photon absorption. The sample with a set of prepared IP-Dip structures was coated by 40 nm thin gold layer. The sample was encapsulated into a prototyped chip with inlet and outlet. The sensing properties were investigated by angular measurement using the prepared solutions of isopropyl alcohol in deionized water of different concentrations. Sensitivity of 478–617 nm per refractive index unit was achieved in angular arrangement at external angle of incidence of 20°.

## 1. Introduction

In recent years, there has been significant attention focused on plasmonic structures thanks to the desirable resonant properties of metal/dielectric interfaces arising from the ability of the concentration of electromagnetic energy at sub-wavelength scale [1,2]. At metal surfaces of plasmonic structures, a collective oscillation of conduction electrons—called surface plasmons (SPs)—as a response to incident electromagnetic wave occurs [3]. The resonance of incident electromagnetic wave with SPs causes the local field enhancement at the metal/dielectric interface leading to optical effect enhancements such as absorption, scattering, transmission, and photoluminescence at resonant frequency [4]. For these aspects, the surface plasmon resonance (SPR) found adequate utilization in fluorescence spectroscopy, Raman scattering, subwavelength imaging, solar cell, and sensing [5,6,7,8]. Due to the high sensitivity at resonance condition, the SPR became crucial for optical sensing of chemical and biochemical substances and physical fields [9,10,11].

SPs have a form of surface electromagnetic wave propagating along the metal/dielectric interface and decaying exponentially perpendicular to the interface in both media. The SPs can be excited optically by an incident electromagnetic wave under phase-matching conditions using special coupling methods. The resonant SP excitation is highly sensitive to the refractive index (RI) change of the surrounding dielectric above the metal surface and is accompanied by an intensity drop of the reflected light. Every slight change of RI on this metal/dielectric boundary leads to a noticeable spectral shift of the resonance wavelength or the resonance angle, so the RI sensitivity is a decisive parameter for the SPR sensors [12].

**Figure 1:**
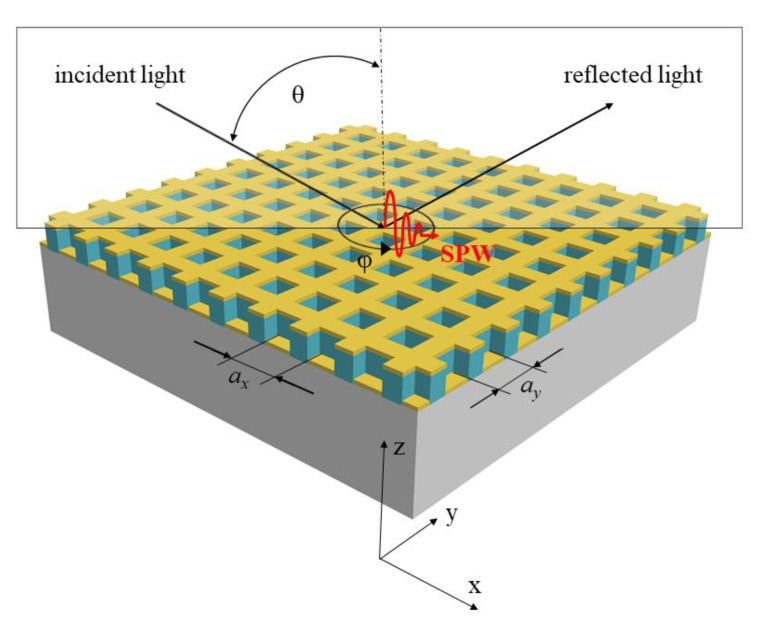
Scheme of the light interaction with the 2D SPR grating structure with plane of incidence oriented across the dominant lines and interpretation of wave vectors.

There are three main techniques for the coupling of incident electromagnetic wave into SP. Prism-based and waveguide-based coupling methods use an evanescent field to excite SP by wave vector matching of evanescent field and that of the SP [13,14]. The grating-based coupling method uses diffraction of light by metallic diffraction grating, which allows incident light to be momentum matched to SP using the additional grating wave vector $k_{g}$ (Figure 1) [15].

SP propagating along metal/dielectric interface has a wave vector $k_{sp}$ higher than the wave vector of incident electromagnetic wave:

(1) $$k_{sp}=\frac{\omega }{c}\sqrt{\frac{\varepsilon_{m}n_{a}^{2}}{\varepsilon_{m}+n_{a}^{2}}}\;,$$

where *ω* is the angular frequency, *c* is the speed of light in vacuum, $\varepsilon_{m}$ is the complex permittivity of metal, and $n_{d}$ is the RI of the analyte. The SP can be then excited by two-dimensional (2D) grating with a square symmetry shown in Figure 1 when the resonance condition is fulfilled:

(2) $$\frac{2\pi }{\lambda }n_{d}\sin\theta +i\frac{2\pi }{\Lambda }+j\frac{2\pi }{\Lambda }=\pm Re\left\{k_{sp}\right\},$$

where λ is the wavelength of the incident light, and θ is the angle of incidence. The wave vector of the light is extended by $k_{g}$ expressed by factor 2π/Λ, where Λ is the period of diffraction grating, and integers *i* and *j* represent the orders of diffraction. The angle *φ* in Figure 1 determines the orientation of 2D SPR grating structure relative to the direction of incident light. By increasing the dimension of the grating structure, the complexity of the plasmonic properties increases too. It has been presented that the application of 2D metallic grating can considerably improve the performance of the sensor [16,17].

The main benefits of SPR-based sensors are real-time and label-free detection. Recently, the grating-based SPR sensors have attracted a great deal of attention thanks to their easy integration possibility into sensing devices [17,18]. In comparison to the other two mentioned coupling techniques, the grating couplers represent a more amenable solution with a simpler setup requirement and lower price for a final sensor [19]. In spite of the fact that it is difficult and still challenging to fabricate metal gratings of high quality and low cost and despite the lower RI sensitivity, which is <1000 nm per refractive index unit (RIU) [16], the grating-based coupling systems are very promising in integrated sensing devices [18]. The SPR grating structures are fabricated by various techniques, such as a laser interference lithography [20,21], nanoimprint lithography [22], electron beam (e-beam) lithography [23,24] and focused ion beam (FIB) [25], then often followed by etching methods. Many of these fabrication methods of gratings consist of several steps, which leads to a longer time required to structure fabrication. There have been presented interesting results of grating-coupled SPR sensors employing grating structures, mainly one-dimensional (1D) [26,27,28,29,30,31]. There is an effort to achieve grating-based sensors with high sensitivity and easy and cheap fabrication. For this reason, many research groups used optical discs as structural template for fabrication of metallic gratings [26,28,32,33,34]. The grating-coupled SPR sensors were used as temperature sensors [21], RI sensors [24,25,33,35] and as biosensors [10,18,19,32,36,37,38,39,40]. Gold-coated polymer gratings brought an efficient way for fabrication of SPR gratings instead of using thick metal layers [29,30,35,41,42,43]. Although many of the presented SPR structures are large-scale, the reduction in their dimensions is needed for microfluidic and lab-on-a-chip (LOC) devices.

Integration of SPR in LOC and microfluidic devices offers the advantages of small volumes, rapid processing, increased reaction rate and enhanced sensing efficiency [44,45]. The integration of sensing and microfluidic system is important to attain a high level of performance using parallel SPR imaging and simultaneous reactions, which increases the throughput of a single chip and provides better delivery of the sample flow. The presented results show integration of the SPR structures into microfluidic chip as 1D gratings [44] and also nanostructured surfaces [45]. These SPR sensors achieved high sensitivity working at low volumes and rapid detection.

Encouraged by these results, we designed and prepared 2D SPR gratings based on IP-Dip polymer for RI sensing of liquid analytes. The 2D polymer grating was prepared using a single-step direct laser writing (DLW) lithography based on the two-photon polymerization (TPP) process. Fabricated 2D polymer gratings were coated by a gold layer with a thickness of 40 nm using a thermal evaporation. The quality of fabricated SPR structures was analyzed by a scanning electron microscope (SEM). The sample with prepared SPR gratings was encapsulated into a prototyped chip prepared by 3D printing and covered by cover glass. The sensing properties were then investigated using prepared liquid analytes with different RI values.

## 2. Design and Fabrication of SPR Structure

**Figure 2:**
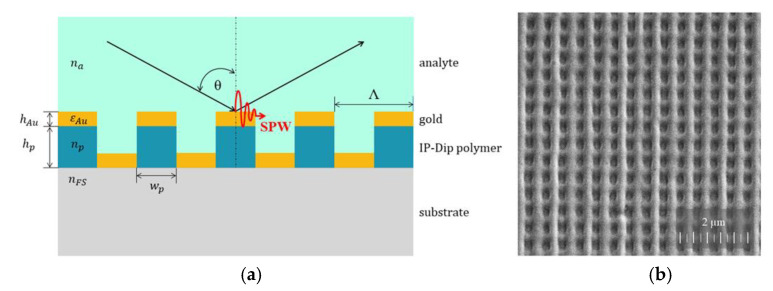
Two-dimensional SPR grating structure: design of the structure (**a**), and SEM image of the structure (**b**).

The SPR structure, whose scheme is shown in Figure 2a, consists of 2D polymer gratings with a square symmetry and a period of Λ = 500 nm coated by a gold layer with a thickness of *h*_Au_ = 40 nm. The expected polymer grating structure parameters in consideration of technology resolution limits are *h*_p_ = 250 nm and *w*_p_ = 250 nm, where *h*_p_ represents the height, and *w*_p_ is the width of the IP-Dip polymer columns. The 2D polymer grating structure was prepared in a square area of dimensions 125 μm × 125 μm via script in program Describe from Nanoscribe GmbH. For the fabrication of polymer gratings, a commercial DLW system Photonic Professional GT from Nanoscribe GmbH was used. The working principle of this system is a non-linear two-photon absorption (TPA) in a negative polymer IP-Dip. This DLW system is equipped with a Ti-sapphire femtosecond laser with a center wavelength of 780 nm, a pulse duration of 100 fs, and a repetition rate of 80 MHz. The polymer grating structures were fabricated using dip-in laser lithography (DiLL) configuration, in which the IP-Dip polymer serves as immersion and photosensitive material at the same time. The femtosecond laser pulses are focused by 63 × 1.4 NA immersion objective in the volume of liquid IP-Dip polymer and TPA initiates the polymerization process in the voxel. We have chosen IP-Dip polymer for fabrication of grating structure because it guarantees ideal focusing and highest resolution for Nanoscribe′s DiLL mode [46,47].

The liquid IP-Dip polymer was deposited as a drop on 0.7 mm thick fused silica glass substrate. The fabrication process was provided by exposure of the IP-Dip photoresist with a scanning speed of 30,000 µm/s and a laser power ranged from 20 mW to 40 mW. After laser writing process, the sample was developed in PGMEA (propylene glycol monomethyl ether acetate) developer, rinsed in isopropyl alcohol, and dried with nitrogen. The sample with a set of prepared IP-Dip structures was coated by a thin gold layer with thickness of 40 nm using a vacuum evaporator (K975X, Quorum Technologies Ltd., Laughton, East Sussex, UK). The thickness of the gold layer was controlled during the thermal evaporation process by a film thickness monitor (10983, Quorum Technologies Ltd., Laughton, East Sussex, UK) with a resolution of 0.1 nm thickness. The quality of fabricated SPR structures was analyzed by a SEM (Tescan, Brno, Czech Republic). The SEM image of a structure detail shown in Figure 2b documents homogeneous 2D grating with a period of Λ = 500 nm with slightly preferred vertical lines given by a two-step process, where the horizontal lines were polymerized first, and vertical lines finished the process. The ellipsoidal-shaped voxel caused the rounded profile of polymerized lines.

## 3. Experimental

The period of Λ = 500 nm was given by our previous experiences and results [47]. A 2D grating structure with this period was prepared close to the resolution limit of DLW system Nanoscribe and showed sufficient quality and homogeneity. The 2D grating structures with periods below 500 nm were of lower quality and were not deep enough, which was manifested in very shallow SPR dips in reflected spectra [47]. Another reason for choosing the period of 500 nm was to achieve the SPR dips in visible (VIS)/ near-infrared (NIR) spectral range with regard to our available detection technique.

**Figure 3:**
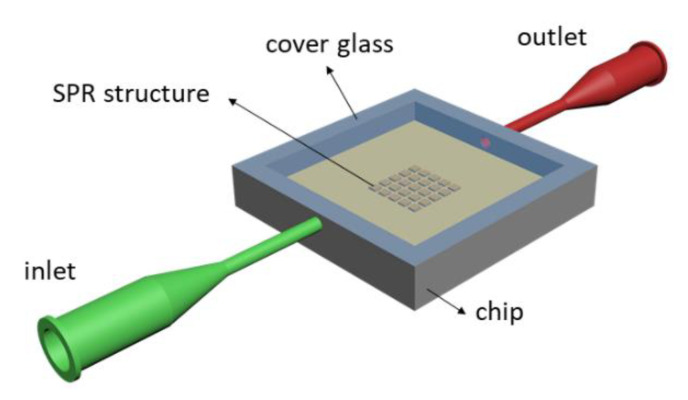
Design of a chip with implemented SPR grating structures for RI measurements of liquid analytes.

The prepared 2D SPR grating SPR structure with a period of Λ = 500 nm was used for RI measuring of the liquids. For the RI measurement of liquid analytes, the prepared 2D plasmonic grating structure was encapsulated into a sensing chip prepared by 3D printing and covered by a cover glass as is shown in Figure 3. The liquid analytes were transported in and out of a sensing chamber of 0.8 mL volume via microfluidic tubings.

To measure the SPR effect and to determine RI sensing ability of the SPR structure, we used different solutions of deionized water (*n* = 1.3330) and isopropyl alcohol (*n* = 1.3753) with isopropyl alcohol concentrations of 20%, 33.33%, 42.86%, and 57.14% volume percent with corresponding refractive indices 1.3470, 1.3552, 1.3605 and 1.3658 measured by an Abbe refractometer (Optika S.r.l., Ponteranica, Italy) at a temperature of 25.5 °C.

**Figure 4:**
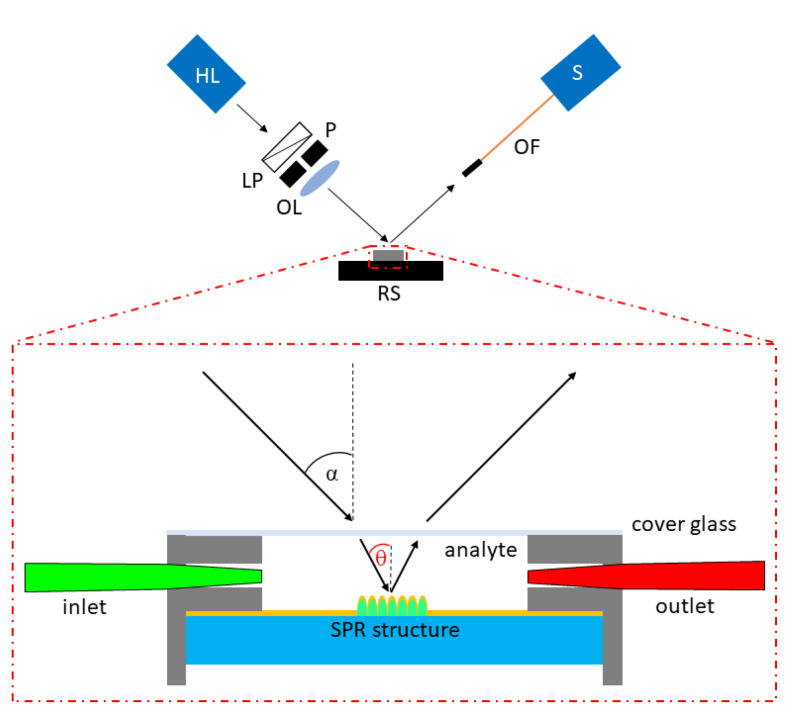
The experimental setup for SPR response measurement with detail of the SPR structure implemented in the chip; HL—halogen lamp, LP—linear polarizer, P—pinhole, OL—objective lens, RS—rotation stage, OF—optical fiber, S—spectrometer.

Figure 4 shows the experimental setup used to measure the SPR response and the RI sensing ability of the 2D grating SPR structure. As a white light source, a halogen lamp was used. The white light was transmitted through a linear polarizer (LPVISE100-A, Thorlabs, Newton, NJ, USA) to achieve *p*-polarization (the vector of electric field is perpendicular to the grating direction). The pinhole was used to obtain a light beam of a small diameter. Using an objective lens (LMPLFLN10X, Olympus, Tokyo, Japan), the light beam was focused on the surface of the SPR structure to achieve a light spot of a 250 μm diameter. The chip with an encapsulated sample was attached on a motorized goniometer stage for selecting the required sample orientation. The reflected light was launched into a spectrometer (USB2000, Ocean Insight, Ostfildern, Germany) by an optical fiber (M76L02, Thorlabs, Newton, NJ, USA). The reflected spectra with SPR dips as a response to RI of different liquid analytes were measured at the external angle of incidence (at the cover glass surface) 20° and 25°. During the measurement, the orientation of the SPR structure was set to be that the preferred grating direction was perpendicular to the plane of incidence of light. The reflectance spectra of the 2D grating SPR structure were normalized by the reference reflectance spectra of a homogenous 40 nm thick gold layer deposited on the same fused silica glass substrate measured at the same values of angles of incidence and RI.

## 4. Results

The SPR-based RI measurements were performed, while the RI in the liquid chip ranged from 1.3330 to 1.3753. The reflectance spectra inducing the SPR effect as a function of the wavelength λ were measured for two external angles of incidence α=20° and α=25°, respectively.

**Figure 5:**
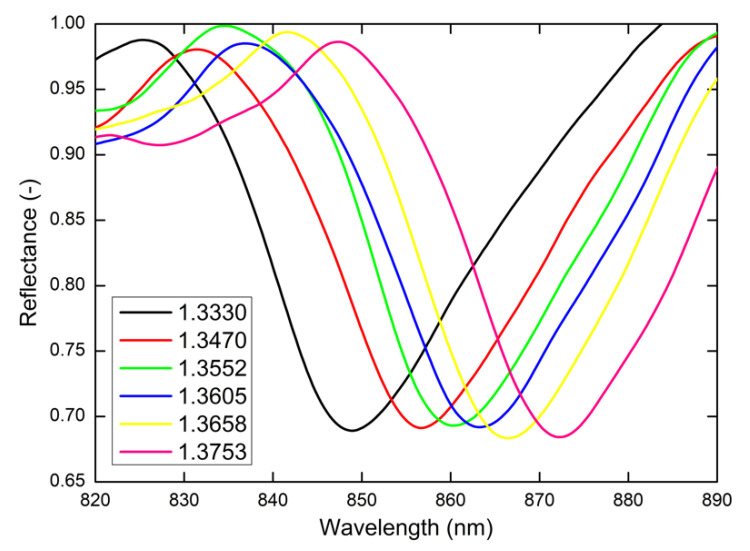
Measured reflectance spectra for the external angle of incidence α=20° and different water/isopropyl alcohol solutions with given values of RI.

**Figure 6:**
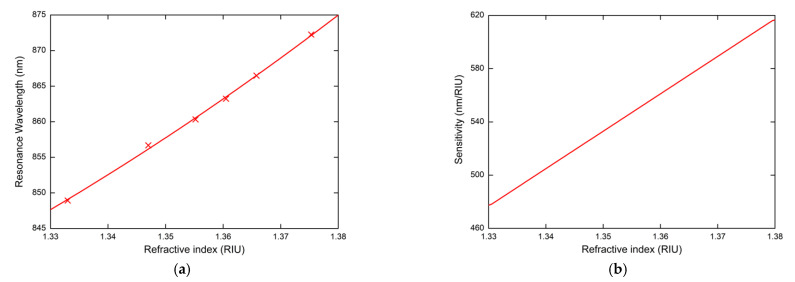
The resonance wavelength with a second-order polynomial fit (**a**), and the sensitivity (**b**) as a function of the refractive index of the liquid analytes for the external angle of incidence α=20°.

Figure 5 shows the measured reflectance spectra for the external angle of incidence α=20° and different water/isopropyl alcohol solutions with the given values of RI. The reflected spectra exhibit well-pronounced SPR dips with almost the same width. The position of the SPR dips corresponding to the resonance wavelength is redshifted in relation to the increasing values of RI. In Figure 6a, the resonance wavelength as a function of RI of the water/isopropyl alcohol solutions with a second-order polynomial fit is summarized. To describe RI sensing properties, it is necessary to designate the RI sensitivity $S_{n}$, defined as the change of the resonance wavelength $\delta \lambda_{r}$ regarding the change in the RI δn ($S_{n}=\delta \lambda_{r}/\delta n$). The resonance wavelength was determined with a precision of 0.01 nm using a zero-crossing in the first derivative of the smoothed reflectance spectrum. The dependence of RI sensitivity $S_{n}$ is linear as is shown in Figure 6b and changes approximately in the range of 478–617 nm/RIU. Another important parameter for quantification of the SPR sensor performance is the figure of merit (FOM), defined as the ratio $FOM=S_{n}/FWHM$, where *FWHM* is the full width at the maximum of the dip. The revealed dips in the reflected spectra show a similar *FWHM* of 26 nm and achieve a value of 24 RIU^−1^.

**Figure 7:**
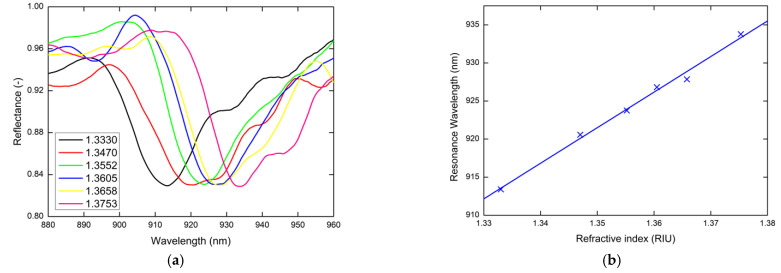
Measured reflectance spectra for the external angle of incidence α=25° (**a**), and the resonance wavelength with a linear fit as a function of the refractive index of the liquid analytes (**b**).

The sensing properties of realized chip with implemented SPR structure were studied also for other angle of incidence, to analyze the effect of experiment geometry on sensitivity parameters. The same experiment was provided for the external angle of incidence α=25°; the measured reflectance spectra are shown in Figure 7a. For this angle, a linear dependence of the resonance wavelength on the refractive index and thus the constant sensitivity $S_{n}$ of 467 nm/RIU was revealed (Figure 7b). Nonlinear to linear change of the resonance wavelength dependence on the RI can be attributed to the evanescent character of the optical field in the liquid analyte. In this case, the FOM ranged from 17 RIU^−1^ to 23 RIU^−1^. For this higher external angle of incidence, the parasitic diffraction affects the final FOM and sensitivity parameters. Similar deterioration of the sensing parameters was also revealed for lower angles. So, the best experimental arrangement with the best sensing properties is at proposed geometry at angle of incidence α=20°.

Many theoretical results have been presented of similarly designed SPR-based RI sensors provided by numerical calculation, but we are focused on the experimental ones. Table 1 summarizes the experimental results of similar grating-coupled SPR-based sensors with wavelength interrogation prepared on substrates with different fabrication methods using gold as a metal. Our proposed RI sensor outperforms some of the RI sensors using thicker gold layers [24,25,38,39,40]. Some of them are prepared by much more technologically- and time-consuming procedures [24,25]. The sensitivity of our RI sensor $S_{n}$ reaches the value of 617 nm/RIU for RIU = 1.38 and for external angle of incidence α=20°. Consales et al. have implemented a 2D polymer/metal SPR grating structure at the end of optical fiber. A disadvantage of the simpler optical setup, which is characteristic for lab-on-fiber (LOF) application in comparison to on-a-chip application, was low RI sensitivity $S_{n}$ = 125 nm/RIU [39]. By employing the IP-Dip polymer grating prepared in a single-step DLW process, we saved a lot of metal material to achieve comparable sensing properties. In addition, our results present a functional sensing device consisting of encapsulated sample with 2D SPR grating structures into a sensing chip. Due to the small size of the prepared SPR structures (125 μm × 125 μm), the structure can be mechanically placed and encapsulated into a LOC device with PDMS (polydimethylsiloxane) channels or implemented into LOF device. Therefore, our presented results favor our SPR structure for LOC and LOF applications.

## 5. Conclusions

We presented fabrication, and characterization of an SPR-based RI sensor consisting of 2D polymer grating structure coated by a thin gold film. The SPR structures were implemented in a simple chip for RI measurements of different water/isopropyl alcohol solutions. The sensing properties were investigated via angular measurement using the prepared solutions of isopropyl alcohol in deionized water. The SPR response of plasmonic structure in visible spectral range with sensitivity of 478–617 nm/RIU for 20° external angle of incidence was achieved. The presented sensor shows an enhanced sensitivity regarding similar grating-coupled SPR-based sensors. The presented results favor this SPR structure for effective RI sensing and document the possibility of use in sensing devices with the possibility of easy LOC and LOF integration.

## Figures and Tables

**Table 1:** Experimental results of similar grating-coupled SPR sensors with gold layers based on wavelength interrogation.

Metal Layer	Fabrication Method	Measured Spectra	Grating	Λ (nm)	RI Range	*S_n_* (nm/RIU)	FOM (RIU^−1^)	Ref.
Ag(80 nm)/Au(2 nm) bilayer	Peeling off + thermal vacuum evaporation	Reflectance	1D	320	1.335–1.365	356	-	[39]
Au(100 nm)	Chemical removing + electron beam deposition	Reflectance	1D	320	1.334–1.3463	425	35	[40]
Au(110 nm)	LIL + thermal evaporation	Reflectance	2D	-	1.333–1.413	465	-	[38]
Au(50 nm)	FIB etching	Transmission	1D	525	1.33303–1.47399	524	-	[25]
Au(500 nm)	e-beam lithography + plasma etching	Reflected	2D	400	1–1.37	544	-	[24]
Au(40 nm)	DLW lithography + thermal evaporation	Reflected	2D	500	1.3330–1.3753	478–617	24	This work
Au(60 nm)	Holographic exposure + sputter coating	Transmission	2D	550	1.330–1.357	648	14	[35]
Au(100 nm)	Soft UV + plasma sputtering	Reflectance	1D	720	1.333–1.398	797	-	[29]
Au(80 nm)	Nanoimprint + plasma sputtering	Reflectance	1D	730	1.333–1.398	800 ± 27	-	[40]

## Data Availability

No new data were created or analyzed in this study. Data sharing is not applicable to this article.
